# Comparative study of the antidiabetic potential of *Paederia foetida* twig extracts and compounds from two different locations in Malaysia

**DOI:** 10.1080/13880209.2019.1610462

**Published:** 2019-06-12

**Authors:** Dai Chuan Tan, Ku Idayu Idris, Nur Kartinee Kassim, Pei Cee Lim, Intan Safinar Ismail, Muhajir Hamid, Rou Chian Ng

**Affiliations:** a Department of Chemistry, Faculty of Science, Universiti Putra Malaysia, Selangor, Malaysia;;; b Department of Microbiology, Faculty of Biotechnology and Biomolecular Sciences, Universiti Putra Malaysia, Selangor, Malaysia

**Keywords:** α-Amylase, α-glucosidase, molecular docking, DPPH, beta-carotene bleaching assay

## Abstract

**Context:***Paederia foetida* L. (Rubiaceae) is an edible plant distributed in Asian countries including Malaysia. Fresh leaves have been traditionally used as a remedy for indigestion and diarrhea. Several phytochemical studies of the leaves have been documented, but there are few reports on twigs.

**Objective:** This study investigates the enzyme inhibition of *P. foetida* twig extracts and compound isolated from them. In addition, *in silico* molecular docking of scopoletin was investigated.

**Materials and methods:** Plants were obtained from two locations in Malaysia, Johor (PFJ) and Pahang (PFP). Hexane, chloroform and methanol extracts along with isolated compound (scopoletin) were evaluated for their enzyme inhibition activities (10,000–0.000016 µg/mL). The separation and identification of bio-active compounds were carried out using column chromatography and spectroscopic techniques, respectively. *In silico* molecular docking of scopoletin with receptors (α-amylase and α-glucosidase) was carried out using AutoDock 4.2.

**Results:** The IC_50_ values of α-amylase and α-glucosidase inhibition activity of PFJ chloroform extract were 9.60 and 245.6 µg/mL, respectively. PFP chloroform extract exhibited α-amylase and α-glucosidase inhibition activity (IC_50_ = 14.83 and 257.2 µg/mL, respectively). The α-amylase and α-glucosidase inhibitory activity of scopoletin from both locations had IC_50_ values of 0.052 and 0.057 µM, respectively.

**Discussion and conclusions:** Separation of PFJ chloroform extract afforded scopoletin (**1**), stigmasterol (**2**) and γ-sitosterol (**3**) and the PFP chloroform extract yielded (**1**), (**2**), (**3**) and ergost-5-en-3-ol (**4**). Scopoletin was isolated from this species for the first time. *In silico* calculations gave a binding energy between scopoletin and α-amylase of −6.03 kcal/mol.

## Introduction

*Paederia foetida* L. (Rubiaceae) is a climbing plant widely distributed in Bangladesh, India, Japan, Malaysia, Myanmar, Nepal, Thailand, Vietnam, Cambodia and China (Ahmed et al. 2014). The plant can grow up to 1500–1800 m high. It gives off a distinctive skunk odour due to the presence of methyl mercaptan (Uddin et al. 2011; Kumar et al. 2014). The common name of *P. foetida* varies from region to region. For instance, the English name is King’s tonic or skunk vine (Nosáľová et al., 2007), whereas in China it is *ji shi teng* and Malaysians call this plant akar sekentut (Osman et al. 2009). In Malaysia, it grows wild in open places and climbs over shrubs or trees. The plant favours humid, sunny regions and is adaptable to different soils. The leaves can be eaten raw or cooked. Malays use the leaves as ‘nasi ulam’ (rice mixed with a variety of chopped herbs) while native communities of Tripura eat the leaves with dry fish (Chanda et al. 2015). The plant has been used to treat toothaches, dysentery, sores, enterosis, enteromagaly, rhinosis, rheumatism, edema, night blindness and digestive problems such as gastritis, diarrhea and ulceration. De et al. (1994) reported *P. foetida* possessed aphrodisiac properties. In addition, it has also been reported to be good for women after childbirth (Upadhyaya 2013). Bioassays show that *P. foetida* exhibits good anti-inflammatory (De et al. 1994), antinociceptive (Hossain et al., 2006), antidiarrheal (Afroz et al. 2006), antioxidant (Upadhyaya 2013; Chanda et al., 2014), antihepatotoxic (Uddin et al. 2011), antidiabetic (Ahmed et al. 2014; Kumar et al. 2014) antitussive (Nosáľová et al. 2007) and gastroprotective (Chanda et al., 2015) activities.

There is clinical evidence that the generation of reactive oxygen species (ROS) which are capable of oxidizing cellular proteins, nucleic acids and lipids, increases in patients with both diabetes and that the onset of diabetes is closely associated with oxidative stress mainly through oxidation, nonenzymatic protein glycation and oxidative degradation of glycated proteins (Sarian et al. 2017). Increase in blood glucose level results in continuous generation of ROS and superoxide anions, which further aggravates diabetic complications by damaging proteins, deoxyribonucleic acid and carbohydrates, leading to an increase in the oxidative stress (Kumar et al. 2013). Antioxidants play an important role in delaying, preventing, scavenging and removing oxidative damage to a target molecule caused by over-peroxidation that may lead to cardiovascular diseases, diabetes, cancer, aging, microbial infections and other conditions (Sheela et al. 2013). The ability of antioxidants to protect against the deleterious effects of hyperglycaemia and also to enhance glucose metabolism and uptake should be considered as a lead alternative in diabetes mellitus treatment (Sarian et al. 2017).

The computational tool, molecular docking can be used to predict noncovalent binding of macromolecules or binding of a macromolecule (receptor) and a small molecule (ligand) (Trott and Olson 2010). Molecular docking predicts the energy profile (binding free energy), strength and stability (binding affinity and binding constant) of complexes using a scoring function (Agarwal and Mehrotra 2016). This method is often utilized to estimate the binding orientation of small molecules to their biomolecular target with the aim for determining their tentative binding parameters. This establishes raw data for rational drug design (structure-based-drug development) of new agents with potentially better efficacy and more specificity (Guedes et al. 2014).

Prashamsa et al. (2017) showed the binding interaction of β-site amyloid precursor protein cleaving enzyme 1 (BACE1) and lupeol using molecular docking. The researchers determined that the hydroxyl group of lupeol formed two hydrogen bonds with the BACE1 catalytic residues, ASP32 (catalytic aspartic residue) and SER35, with a binding energy of −8.63 kcal/mol for BACE1 (PDB ID: 2WJO). The van der Waals interaction of lupeol with TYR71, GLN73, TRP76, LYS107, PHE108 and ILE118 further stabilized the enzyme–inhibitor interaction.

In this study, antidiabetic properties of different Malaysia *P. foetida* twig extracts from two different locations in Malaysia namely Johor (PFJ) and Pahang (PFP) are compared in order to determine the location that provides the most active sample to enable further harvesting essential for an extensive study of the plant. The isolation of phytoconstituents and *in silico* molecular docking of the isolated compounds with the receptors (α-amylase and α-glucosidase) were carried out. Part of the evaluation of their enzyme inhibition activity and the antioxidant activity of the plant extracts are reported.

## Materials and methods

### Plant materials

*P. foetida* was collected from two different locations in Malaysia from Temerloh, Pahang (East Coast) and Ledang, Johor (Southern) on 1 October 2015 and 7 June 2017, respectively. Both plant samples were submitted to the Institute of Bioscience (IBS), UPM Serdang for plant identification by Dr. Mohd. Firdaus Ismail from the Biodiversity Unit. They were given specimen voucher numbers SK 2908/15 and SK3177/17, respectively. The twigs of the plants were dried at room temperature and ground into powder.

### Chemicals and reagents

The α-amylase and α-glucosidase enzymes were purchased from Megazyme. *p*-Nitrophenyl α-d-glucopyranoside, soluble starch, potassium sodium tartrate, 3,5-di-nitro salicylic acid (DNS), sodium hydroxide, dimethyl sulfoxide (DMSO), 2,2-diphenyl-1-picrylhydrazyl (DPPH), β-carotene, linoleic acid, butylated hydroxytoluene (BHT), butylated hydroxyanisole (BHA), vitamin C (ascorbic acid), vitamin E (α-tocopherol), Follin–Ciocalteau reagent, gallic acid and other chemicals and solvents of analytical grade were purchased from Sigma (MO, USA). Gravity flow chromatography columns were using Merck Kieselgel 60 F_254_ Art No 1.07734.1000 (63–200 µm) for fractionation process and 1.09385.1000 (40–63 µm) for isolation.

### General procedures

The melting point of scopoletin was determined using the Barnstead Electrothermal IA 9000 series digital melting point apparatus equipped with a microscope. Perkin Elmer Fourier-Transform Infrared (FTIR) spectrophotometer which applied Universal Attenuated Total Reflection (UATR) was used to determine infrared spectra of the samples. The infrared spectra of the sample were observed in the range 280–4000 cm^−1^. The mass spectrum of the compound was recorded by using a Shimadzu Gas Chromatography–Mass Spectrometer (GC–MS) model QP5050A with BPX5 (5% phenylmethylsilane) capillary column (30 × 250 μm × 0.25 μm). A JEOL Fourier-Transform Nuclear Magnetic Resonance (FT-NMR) spectrometer was used to determine proton (^1^H) and carbon-13 (^13^C) Nuclear Magnetic Resonance (NMR) spectra of the compound. ^1^H NMR was measured at 500 MHz while ^13^C NMR was measured at 125 MHz. Two-dimensional (2D) NMR including correlated spectroscopy (COSY), heteronuclear multiple bond connectivity by heteronuclear multiple bond correlation (HMBC) and heteronuclear multiple quantum correlation (HMQC) were also done. The antidiabetic and antioxidant assays were performed using the µ-QUANT model microplate reader.

### General extraction and isolation

The extraction of *P. foetida* twigs was carried out following the successive organic solvent extraction method (Yeap et al. 2017) with slight modification. Firstly, the powdered twigs (1.2 kg) were sequentially soaked for 72 h in four solvents of different polarity (hexane, chloroform, ethyl acetate and methanol) using the cold maceration method. Next, the sample was filtered and concentrated using rotary vacuum evaporation at 40 °C giving four different crude extracts. PFJ hexane extract (10.10 g) was yellow, the chloroform extract a dark green solid (11.75 g), the ethyl acetate extract a dark green semi-solid (4.16 g) and the methanol extract a dark brown gummy (79.34 g). Hexane (7.07 g), chloroform (8.55 g), ethyl acetate (2.04 g) and methanol (22.31 g) extracts of PFP were of similar color to those obtained from PFJ. All extracts were subjected to α-amylase and α-glucosidase inhibition assays. The most active extract chloroform for both PFJ and PFP was further subjected to column chromatography technique in an attempt to isolate the bioactive compounds.

#### Isolation of bioactive compound from chloroform extract PFJ

Seventeen fractions of 100 mL each were collected from the fractionation of PFJ chloroform extract. The fractions were collected and combined based on similarity in their Thin Layer Chromatography (TLC) profiles. A total of four fractions were obtained. Fraction 2 was chromatographed with the solvent mixture of hexane, ethyl acetate and methanol as eluents to give 18 subfractions (100 mL/fraction). Subfraction 15 was further subjected to gravity column chromatography with the solvent mixture hexane, ethyl acetate and methanol as eluents to yield a total of eight sub-subfractions. The sub-subfraction 4 yielded 8.2 mg of scopoletin (**1**). To our knowledge, this is the first report of isolation of scopoletin from *P. foetida*. Repeated chromatographing of sub-fractions six with hexane–ethyl acetate afforded a mixture (3 mg) of stigmasterol (**2**) and γ-sitosterol (**3**).

#### Isolation of bioactive compound from chloroform extract PFP

Fractionation of chloroform extracts PFP gave 50 fractions of 100 mL each. The fractions were collected and combined based on similarity in their TLC profiles. A total of eight fractions were obtained. Fractions 5, 6 and 7 were purified by silica gel column chromatography. Elution of fraction 5 with hexane–ethyl acetate afforded a mixture of compounds (2 mg), stigmasterol (**2**), γ-sitosterol (**3**) and ergost-5-en-3-ol (**4**). Further purification on fraction 6 with hexane–ethyl acetate yielded 1 mg of scopoletin (**1**) as white solid. Unfortunately, fraction 7 did not give any pure compounds due to its small amount.

### Enzymatic assays

Antidiabetic activity of the extracts and isolated materials were evaluated through two enzymatic inhibition assays namely α-amylase and α-glucosidase.

#### α-Amylase inhibition

The degree of α-amylase inhibition was determined using a slight modification of the iodine-starch test (Ee Shian et al. 2015). The total volume of sample consisted of 60 μL 0.1 M sodium phosphate buffer, 20 μL α-amylase (1 U/mL) and 50 μL extract (0.0078–0.5 mg/mL). The mixture was incubated for 15 min at 37 °C. Soluble starch (0.5%) (50 μL) was then added to the sample, and the mixture was incubated once again. The enzyme activity was stopped by the addition of 20 μL 1 M hydrochloric acid followed by incubation in a hot water bath for 5 min. Lastly, iodine reagent (50 μL) was added to the mixture before the absorbances were measured at 620 nm. Acarbose and distilled water were used as positive and negative controls. α-Amylase enzyme activity was determined using the formulae:

$$\begin{array}{c} \text{Relative}\;\alpha -\text{amylase}\;\text{enzyme}\;\text{activity}\;(\%)=(\text{enzyme}\;\text{activity}\;\text{of}\;\text{sample}/\text{enzyme}\;\text{activity}\;\text{of}\;\text{negative}\;\text{control})\;\times 100\%\\ \alpha -\text{Amylase}\;\text{enzyme}\;\text{inhibition}\;(\%)=100\%-\text{Relative}\;\alpha -\text{amylase}\;\text{enzyme}\;\text{activity}\;(\%) \end{array}$$

The IC_50_ values were determined from the plot of α-amylase inhibition against concentration.

#### α-Glucosidase inhibition

α-Glucosidase inhibition assay was performed using a slight modification of the procedures outlined by Collins et al. (1997) and Deutschländer et al. (2009). Substrate solution (1.0 mM) was prepared by dissolving 0.006 g of *p*-nitrophenyl-α-d-glucopyranoside (PNPG) in 20 mL of 50 mM phosphate buffer (pH 6.5). Then, the working enzyme solution (2 µL/mL) was prepared by diluting 2 µL of stock α-glucosidase solution in 998 µL of 50 mM phosphate buffer. Test samples were prepared in dimethyl sulfoxide (DMSO), and six serial dilutions were performed. The concentrations for the four crude samples were 10,000 µg/mL. Test sample (10 µL), 130 µL of 30 mM of phosphate buffer solution and 10 µL of α-glucosidase working solution were mixed in a 96-well microplate and pre-incubated at 37 °C for 30 min. After pre-incubation, 50 µL of PNPG was loaded into the wells, and the reaction mixtures were incubated for another 30 min at 37 °C. The reaction was quenched by adding 50 µL of 2 M glycine (pH 10). The absorbance of each reaction was recorded at 405 nm. The α-glucosidase inhibition activity of the test samples was expressed as the percentage of inhibition and calculated as follows:

$$\%\;\text{Inhibition}\;=\;\left(\Delta A_{c}\;-\;\Delta A_{e}/\Delta A_{c}\right)\;\times \;100\%$$

where Δ*A*_c_ is the difference in absorbance between the negative control with enzyme and the blank negative control (without enzyme) while Δ*A*_e_ is the absorbance difference between the test sample with enzyme and the blank test sample (without enzyme). The negative control was conducted in the same way by replacing the test sample with DMSO. For the blank negative control and blank test sample, the enzyme solution was replaced by 30 mM phosphate buffer solution.

### *In silico* molecular docking

The crystal structure of the receptor molecules, α-amylase (PDB ID: 4W93) and α-glucosidase (PDB ID: 3WY1) were downloaded from the protein databank. Molecular docking was performed using AutoDock (Trott and Olson 2010). Extra or unwanted chains, crystal water and non-polar hydrogen atoms were removed from the receptor, and hydrogen atoms were added. The receptor structures obtained were used to continuously clean the structure of MGAM protein using text editor software. Minimized structures were subjected to docking studies. The molecular structure of the ligand or isolated compound was saved in MOL file from ChemDraw. The Gasteiger charges and hydrogen atoms were added to the ligand or isolated compound using AutoDock Tools. Docking Grid Box was used for calculating the grid maps and centred on the ligand coordinate. Docking Grid Box has dimensions of 28 Å × 28 Å × 28 Å, centred on the ligand or isolated compound with a grid spacing of 1 Å. The docking results were visualized and analyzed using Discovery Studio Visualizer software (DA, USA).

### Antioxidant assays

Antioxidant activities of *P. foetida* extracts and the isolated compound were evaluated by DPPH free radical scavenging and β-carotene bleaching (BCB) following methods described by Yeap et al. (2017).

#### 2,2-Diphenyl-1-picrylhydrazyl (DPPH) free radical scavenging

Methanol DPPH solution (300 μM) and sample solution with concentration of 15.6-1000 µg/mL were prepared. Methanol DPPH solution (30 μL) was added to each 70 μL sample solution in a 96-well microplate. Then, the mixture was incubated in the dark for 30 min. Then, the colour changes of the mixture from purple to yellow was measured at 517 nm using an ELISA reader. Vitamin E solutions (prepared different concentrations) were used as standards. The ability of the sample to scavenge DPPH radicals was calculated by using the formula:

$$\%\;\text{Inhibition}\;=\;1\;-\;\left({\text{OD}}_{\text{sample}}/{\text{OD}}_{\text{blank}}\right)\;\times \;100\%$$

OD_blank_ is the absorbance of blank; OD_sample_ is the absorbance of the reaction mixture.

A graph of percentage inhibition against concentration was plotted to obtain the IC_50_ value. The average was calculated using three replicates.

#### β-Carotene bleaching (BCB)

Solutions (1 mg/mL) of samples and of α-tocopherol were prepared in analytical grade methanol, while a 1 mg/mL solution of β-carotene was prepared in chloroform. A 210 μL of β-carotene solution was pipetted into round-bottomed flask containing 5 μL of linoleic acid and 42 μL Tween 20. The chloroform was then removed from the mixture using rotary evaporation. A 10 mL of distilled water was then added to the dried mixture and shaken to form an emulsion. A 200 μL of the emulsion was pipetted into 96-well microplate containing 50 μL of samples and α-tocopherol. The absorbance of mixtures at the initial time (*t* = 0) and after 2 h (*t* = 2) was read at 470 nm. The plate was incubated in an oven at 40–50 °C for 2 h. The sample having antioxidant properties showed a decrease in absorbance which was measured after every 30 min. The percentage antioxidant activity was calculated as follows:

$$\text{Antioxidant}\;\text{activity},\;\text{AA}\%\;=1\;-\;\left[\left(A_{t}\;=\;0\;–\;A_{t}\;=\;2\right)/\left(A_{c}=\;0\;–\;A_{c}=\;2\right)\right]\;\times \;100\%$$

where *A*_t_ is sample; *A*_c_, control.

### Statistical analysis

All results are expressed as mean ± SE and differences between means were statistically analyzed using the *t*-test for comparison between two treatments. *p* < 0.05 was considered significant.

## Results and discussion

### Isolation and identification of phytochemicals

**Figure 1.**
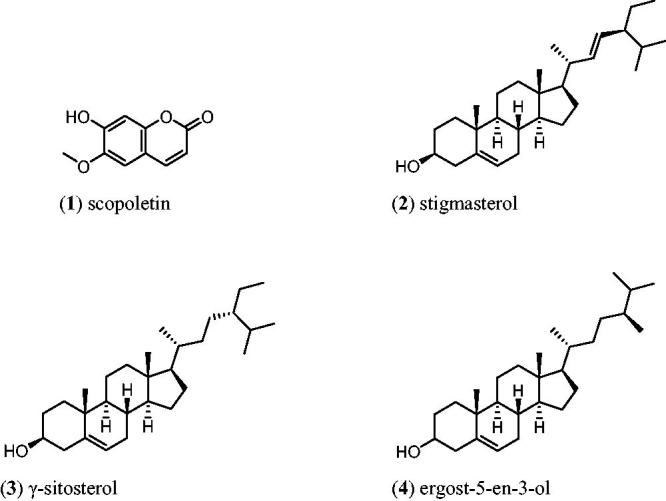
The chemical compounds found in *Paederia foetida*.

The NMR and GC–MS spectral data for compound **1** were found to be in agreement with the literature data while compounds **2**, **3** and **4** were identified based upon their GC–MS analysis which gave molecular ion peaks at *m*/*z* 400 [M^+^], 412 [M^+^] and 414 [M^+^] corresponding to stigmasterol (**2**), γ-sitosterol (**3**) and ergost-5-en-3-ol (**4**), respectively (see Figure 1). Pierre and Moses (2015) have noted that stigmasterol is quite difficult to isolate in its pure form; it is always mixed with β-sitosterol. In addition, the *R*_f_ index for both compounds are the same even though different solvent system were used.

### Antidiabetic activities

**Figure 2.**
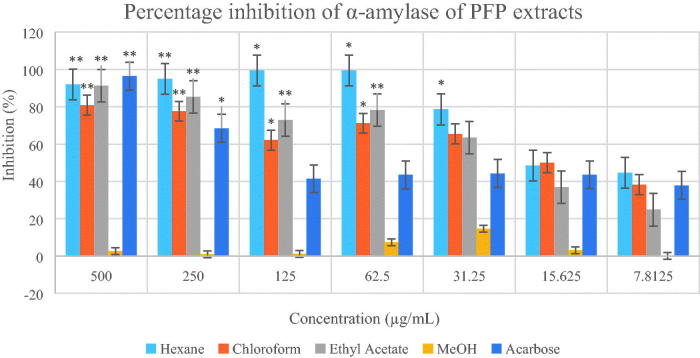
Percentage inhibition of α-amylase by different extracts of *Paederia foetida* twig from Pahang, Malaysia. The different extracts were compared with acarbose and *p* < 0.05 (*p* = 0.0001). *indicates significance at the 0.05 level, **indicates significance at the 0.01 level.

**Figure 3.**
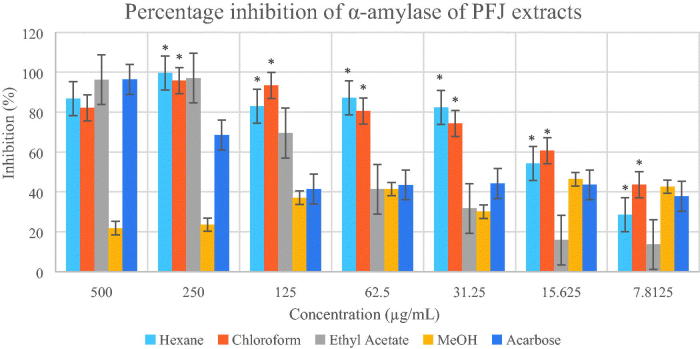
Percentage inhibition of α-amylase by different extracts of *Paederia foetida* twig from Johor, Malaysia. The different extracts were compared with acarbose and *p* < 0.05 (*p* = 0.0101). *indicates significance at the 0.05 level.

**Figure 4.**
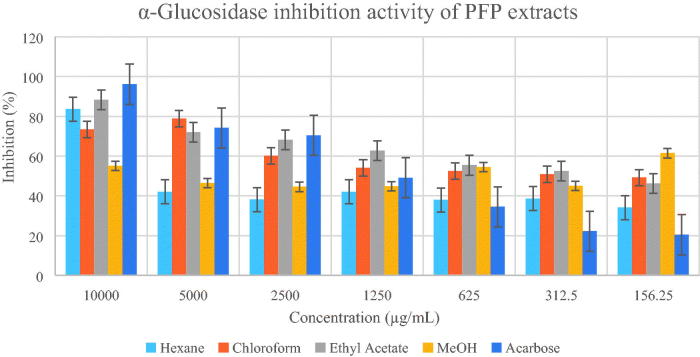
Percentage inhibition of α-glucosidase by different extracts of *Paederia foetida* twig from Pahang, Malaysia. The different extracts were compared with acarbose and *p* < 0.05 (*p* = 0.0080).

**Figure 5.**
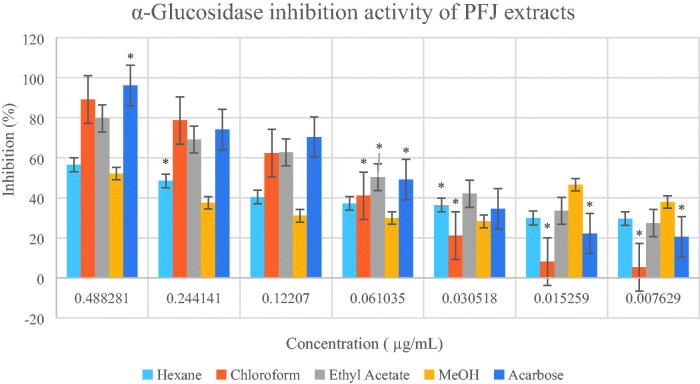
Percentage inhibition of α-glucosidase by different extracts of *Paederia foetida* twig from Johor, Malaysia. The different extracts were compared with acarbose and *p* < 0.05 (*p* = 0.0019). *indicates significance at the 0.05 level.

Based on the two enzymatic inhibition assay models, PFJ chloroform extract showed the highest antidiabetic activity. The IC50 values and inhibitory effects (%) are given in Table 1 and Figures 2–5. PFJ chloroform extract inhibited α-amylase activity with an IC_50_ value of 9.60 ± 0.01 µg/mL which is comparable to standard acarbose (16.37 ± 0.01 µM). The inhibitory potential of the PFJ chloroform extract was significantly different (*p* < 0.05) from that of other extracts. The PFJ chloroform extract also showed the strongest inhibition on α-glucosidase enzyme among all the extracts with IC_50_ of 245.6 ± 0.01 µg/mL and *p* < 0.05. The isolated compound, scopoletin, showed good α-amylase and α-glucosidase inhibition activity with the IC_50_ values of 0.052 ± 0.13 and 0.057 ± 0.011 µM, respectively. The PFP chloroform extract showed inhibition of α-amylase activity at the concentration ranging from 500 to 62.5 µg/mL (Figure 2) whereas the PFJ chloroform extract displayed stronger inhibition with a lower concentration ranging from 250 to 7.81 µg/mL (Figure 3). In α-glucosidase inhibition, the PFJ chloroform extract showed a significant difference (*p* < 0.05) (Figure 4), however no significant difference found in the PFP chloroform extract (Figure 5).

**Table 1.** IC_50_ values for α-glucosidase and α-amylase inhibition by extracts of *Paederia foetida* twig.

Samples	α-Glucosidase inhibition activity IC_50_ (μg/mL)		α-Amylase inhibition activity IC_50_ (μg/mL)	
	Pahang (PFP)	Johor (PFJ)	Pahang (PFP)	Johor (PFJ)
Hexane	2394 ± 0.02^abcd^	1207 ± 0.03^abce^	11.64 ± 0.02^a^	13.80 ± 0.01^b^
Chloroform	257.2 ± 0.01	245.6 ± 0.01^abcf^	14.83 ± 0.05	9.60 ± 0.01^c^
Ethyl acetate	285.1 ± 0.01	1062 ± 0.01	23.33 ± 0.02	62.08 ± 0.00
Methanol	1472 ± 0.06^a^	>10 000	>500	>500
Acarbose	0.056 ± 0.01 (0.087 ± 0.01 μM)		10.57 ± 0.01 (16.37 ± 0.01 μM)	
Scopoletin	0.011 ± 0.11^defg^ (0.057 ± 0.11 μM)		0.010 ± 0.13^abcg^ (0.052 ± 0.13 μM)	

All values are expressed as mean ± standard deviation (*n* = 3). *p* < 0.05. Data with different superscripts (a, b, c, d, e, f, g) were considered significant.

The α-amylase inhibition activity is in the order scopoletin > PFJ chloroform extract > acarbose > PFP hexane extract > PFJ hexane extract > PFP chloroform extract > PFP ethyl acetate extract > PFJ ethyl acetate extract, whereas the α-glucosidase inhibition activity is in the order scopoletin > acarbose > PFJ chloroform extract > PFP chloroform extract > PFP ethyl acetate extract > PFJ ethyl acetate extract > PFJ hexane extract > PFP methanol extract > PFJ hexane extract.

α-Amylases commonly occur in plants, animals and microorganisms. α-Amylase plays an important role in the catalytic hydrolysis of α-1,4-glucosidic linkages in starch, glycogen and in several oligosaccharides (Bhandari et al. 2008). α-Amylase is involved in the breakdown of long-chain carbohydrates while α-glucosidase breaks down simple sugars and disaccharides to glucose. Inhibition of these enzymes can help to reduce the rate of carbohydrate digestion, delay intestinal absorption and slow down the sharp rise in blood sugar levels that diabetic patients typically experience after meals (Ee Shian et al. 2015).

α-Glucosidase breaks down disaccharides in carbohydrate hydrolysis to release simpler, absorbable sugars, monosaccharides. Lebovitz (1998) stated that α-glucosidase inhibitors are competitive inhibitors that delay the digestion of complex carbohydrates and hydrolyze oligosaccharides into monosaccharides. This reduces a rise in postprandial plasma glucose. Due to their pharmacological action, α-glucosidase inhibitors also cause a concomitant decrease in postprandial plasma insulin and gastric inhibitory polypeptide and a rise in late postprandial plasma glucagon-like peptide 1 levels. Venable and Aschenbrenner (2005) also mentioned that the inhibition of this enzyme system reduces the rate of digestion of carbohydrates. This results in less glucose being absorbed because the carbohydrates are not broken down into glucose molecules. In diabetic patients, the short-term effect of drug therapies is to decrease current blood glucose levels whereas the long-term effect is a small reduction in hemoglobin_A1c_ level.

The amount of sugar available for absorption is lowered by the inhibition of these two enzymes during digestion; thus, it can be used as a good strategy in the early treatment of type 2 diabetes mellitus. Enzyme inhibitors such as acarbose aid in minimizing the activity of amylase and glucosidase at the brush border of the small intestine, restricting the breakdown of starch and sucrose, delaying the digestion complex carbohydrates and absorption of glucose in the alimentary tract, finally slowing down the rise of glucose in the blood after a meal (Ee Shian et al. 2015).

The difference in inhibition activities and variation of chemical compounds present in Pahang and Johor extracts may be due to weather conditions such as rainfall. There are many examples where rainfall affects the chemical of the plant extracts and their bioactivity. Santos et al. (2016) reported that the amounts of pogostol and α-yalangene in *Hortia oreadica* were dependent on weather conditions. Ozer et al. (2017) stated that rainfall is an important factor affecting the chemical compositions and bioactivity of the oils from *Chenopodium botrys*. Pahang is situated on the East Coast of Malaysia facing the Northeast Monsoon whereas Johor faces the Southwest Monsoon (Southern Malaysia). Suhaila et al. (2010) reported the total rainfall was found to have decreased during the Southwest monsoon season. In contrast, an increasing trend in the total amount of rainfall was detected during the Northeast monsoon period. The Northeast Monsoon brings more rain than the Southwest. The east coast of Malaysia is generally more prone to annual flooding than is the southern area. This can cause plants to become stressed and even wilt due to the presence of excessive amounts of water.

The results of this study display the PFJ chloroform extract as a potential carbohydrate hydrolyzing enzyme inhibitor because it is as effective as acarbose in α-amylase inhibition activity. The isolated compound, scopoletin could be responsible for the activity of this chloroform extract. Since the binding interaction of scopoletin as a carbolytic enzyme inhibitor has not yet been investigated, the determination of interactions was investigated by molecular docking.

### Computational docking study

The determination of the mechanism of inhibition between receptors (α-amylase and α-glucosidase) and scopoletin was performed using AutoDock Tools. The docked conformations of receptor–ligand complexes generated were analyzed. Hydrogen bonding and hydrophobic interactions from α-amylase and α-glucosidase with scopoletin were visualized and analyzed by Discovery Studio Visualizer. Figure 8 shows molecular docking results for the binding interaction between α-amylase and scopoletin. The α-amylase-scopoletin complex showed lower energy (−6.03 kcal/mol) with better binding affinity compared to other α-amylase–scopoletin complexes generated. Selected inhibitor complex was analyzed for geometrical parameters such as hydrogen bonding, hydrophobic interactions and amino acid residues. GLY139 and GLU171 catalytic residues interacted with scopoletin through hydrogen bonding with bond distances of 3.32 and 4.75 Å, respectively. The π-alkyl bond that interacted with ALA169 and the aromatic ring of scopoletin has bond distance of 5.99 Å. In addition, some hydrophobic interactions were also observed between scopoletin, ASN137, ASP138, GLY133, LYS140, LYS172, PHE136 and TRP134 residues.

The α-glucosidase-scopoletin inhibitor complex showed lower energy (−2.92 kcal/mol) with better binding infinity compared to other α-glucosidase–scopoletin complexes generated. Two conserved residues, ARG450 and GLN439, from the glucosidase family showing hydrogen bonding to the ether group of scopoletin at distance of 6.67 and 5.55 Å, respectively, were reported using Discovery Studio Visualizer (Figure 9). TYR41 showed a π–π bond interaction with the aromatic ring of scopoletin. Moreover, five amino acid residues, ASP40, LYS38, SER44, TRP409 and VAL435, showed hydrophobic interactions with scopoletin. The α-amylase–scopoletin inhibitor complex showed stronger inhibition activity than α-glucosidase–scopoletin inhibitor complex due to lower binding energy, a higher number of closest amino residues and lower root-mean-square deviation (RMSD). All the interactions are important for the positioning of scopoletin in the pocket to inhibit the activity of α-amylase and α-glucosidase. This interaction may provide further stability to the α-amylase–scopoletin complex compared to the α-glucosidase–scopoletin complex (Table 3). The scopoletin therefore has the potential to be an antidiabetic inhibitor lead compound.

### Antioxidant activities

**Figure 6.**
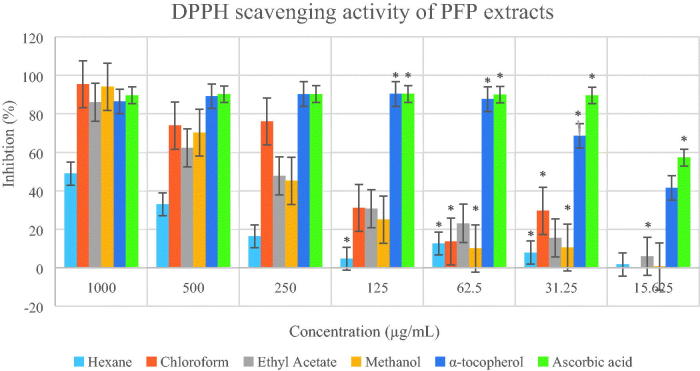
Percentage inhibition of DPPH scavenging activities by different extracts of *Paederia foetida* twig from Pahang, Malaysia. The different extracts were compared with α-tocopherol and ascorbic acid, *p* < 0.05 (*p* = 0.0021). * indicates significance at the 0.05 level.

**Figure 7.**
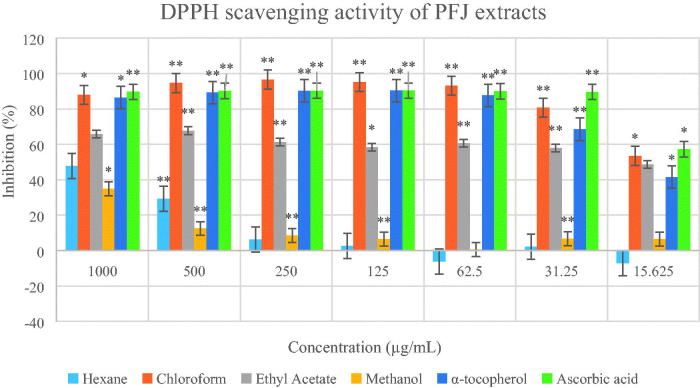
Percentage inhibition of DPPH scavenging activities by different extracts of *Paederia foetida* twig from Johor, Malaysia. The different extracts were compared with α-tocopherol and ascorbic acid, *p* < 0.05 (*p* = 0.0001). * indicates significance at the 0.05 level, ** indicates significance at the 0.01 level.

The IC_50_ values of all crude extracts (Table 2) were calculated using the graph of DPPH percentage inhibition against concentration for each of the extracts (Figures 6 and 7). The PFJ chloroform extract showed the strongest scavenging effect followed by the PFJ ethyl acetate extract, and the PFP chloroform extract with the IC_50_ values of 27.27 ± 0.01, 35.85 ± 0.05 and 181.97 ± 0.07 µg/mL, respectively. α-Tocopherol and ascorbic acid are natural antioxidants which show potent scavenging effect with the IC_50_ values of 44.32 ± 0.00 and 86.02 ± 0.01 µM, respectively. The *t*-test performed on DPPH inhibition (%) at different concentrations of PFJ chloroform extract was significantly different (*p* < 0.01) whereas the PFP chloroform extract was not significantly inhibited.

**Figure 8.**
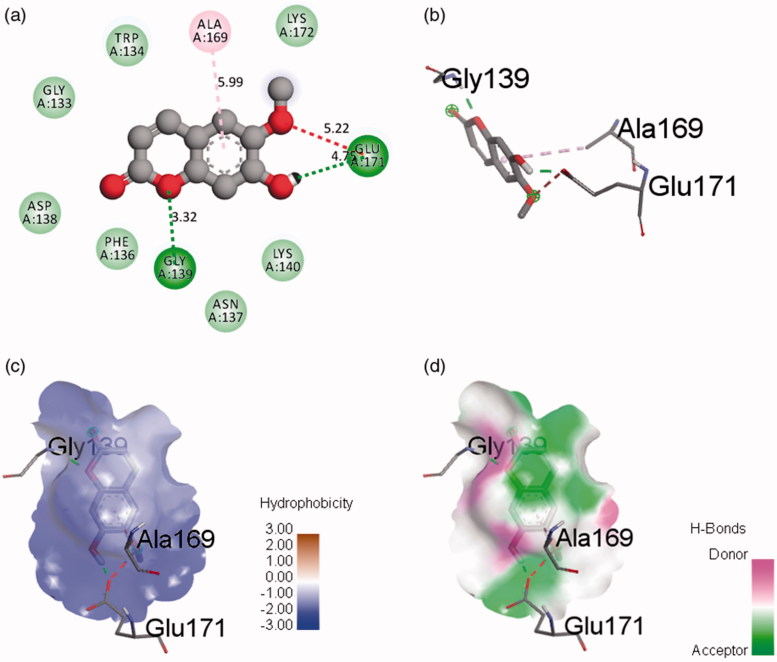
Binding of scopoletin to α-amylase pocket. (a) 2D-diagram of ligand interaction. (b) receptor–ligand interaction. (c) Hydrophobic surface. (d) Hydrogen bond surface.

**Figure 9.**
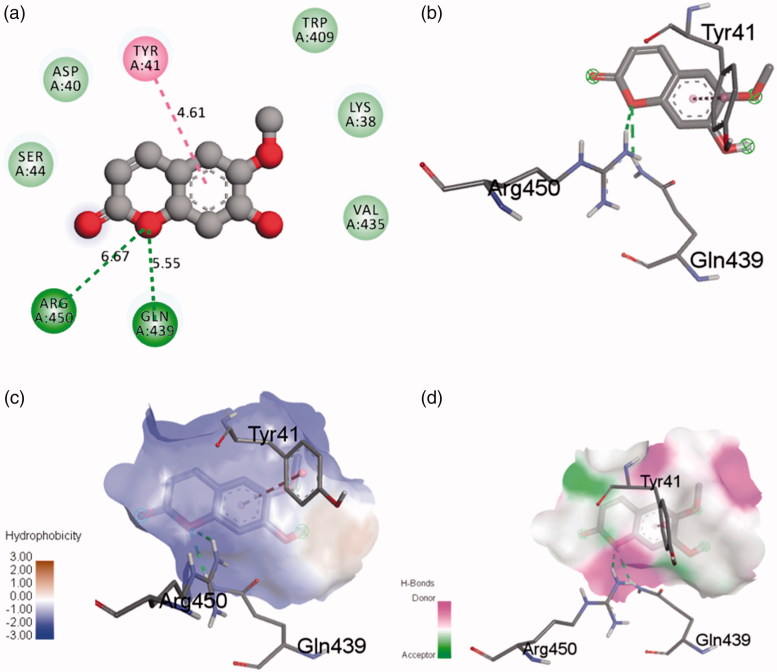
Binding of scopoletin to α-glucosidase pocket. (a) 2D-diagram of ligand interaction. (b) receptor–ligand interaction. (c) Hydrophobic surface. (d) Hydrogen bond surface.

**Table 2.** β-Carotene bleaching activity and IC_50_ values of DPPH by extracts of *Paederia foetida* twig.

Samples	DPPH IC_50_ (μg/mL)		β-Carotene bleaching activity, %	
	Pahang (PFP)	Johor (PFJ)	Pahang (PFP)	Johor (PFJ)
Hexane	>1000	>1000	88.08 ± 0.02^a^	69.41 ± 0.01^e^
Chloroform	181.97 ± 0.07^b^	27.27 ± 0.01^abdef^	92.24 ± 0.02^b^	72.89 ± 0.03^abcdef^
Ethyl acetate	>1000	35.85 ± 0.05^e^	80.07 ± 0.02^c^	73.83 ± 0.01^e^
Methanol	>1000	>1000	77.14 ± 0.02^d^	77.66 ± 0.01^fg^
α-Tocopherol	19.09 ± 0.00^abegh^ (44.32 ± 0.00 μM)		99.35 ± 0.02^abcdegh^	
Ascorbic acid	15.15 ± 0.01^abegi^ (86.02 ± 0.01 μM)		n.a.	
BHT	9.97 ± 0.00^aegj^ (45.25 ± 0.00 μM)		95.88 ± 0.01^abcdegj^	
BHA	10.42 ± 0.01^abegk^ (57.81 ± 0.01 μM)		98.99 ± 0.00^abcdegk^	
Scopoletin	655.5 ± 0.00^fhijk^ (3411.21 ± 0.00 μM)		71.73 ± 0.01^fhijk^	

All values are expressed as mean ± standard deviation (*n* = 3). *p* < 0.01. Data with different superscripts (a, b, c, d, e, f, g, h, i, j, k) were considered significant.

n.a. are not applicable.

**Table 3.** Binding interaction of the complex ligands.

Complex ligand	Binding energy (kcal/mol)	Number of closest residues	RMSD (Å)
α-Amylase–scopoletin	−6.03	7	3.16
α-Glucosidase–scopoletin	−2.92	5	6.04

The β-carotene bleaching assay indicated that PFP chloroform extract has the highest antioxidant activity (92.24 ± 0.02%) which is comparable to α-tocopherol (Table 2). The β-carotene bleaching activity of PFJ is in the order: α-tocopherol > BHA > BHT > methanol extract > ethyl acetate extract > chloroform extract > hexane extract. In addition, the β-carotene bleaching activity of PFP is in the order: α-tocopherol > BHA > BHT > chloroform extract > hexane extract > ethyl acetate extract > methanol extract.

## Conclusions

The PFJ chloroform extract showed the strongest inhibitory activity toward α-amylase. The chloroform extract of PFP afforded scopoletin (**1**), stigmasterol (**2**), γ-sitosterol (**3**) and ergost-5-en-3-ol (**4**) while that from PFJ gave (**1**), (**2**) and (**3**). The *in silico* assessment of scopoletin as an antidiabetic agent indicated that the α-amylase–scopoletin complex has good stability with a binding affinity of −6.04 kcal/mol. *P. foetida* can be considered as a potential resource for natural herbal antidiabetic drugs which can serve as an alternative to synthetic oral hypoglycaemic drugs with minimum side effects.
